# The effect of neostigmine on postoperative delirium after colon carcinoma surgery: a randomized, double-blind, controlled trial

**DOI:** 10.1186/s12871-022-01804-4

**Published:** 2022-08-22

**Authors:** Fanghao Liu, Xu Lin, Yanan Lin, Xiyuan Deng, Yuwei Guo, Bin Wang, Rui Dong, Yanlin Bi

**Affiliations:** 1 Department of Anesthesiology, Qingdao Municipal Hospital Affiliated to Qingdao University, Qingdao, China; 2 Department of Anesthesiology, Nanjing Drum Tower Hospital, Nanjing, China

**Keywords:** Postoperative delirium, Neostigmine, Colon carcinoma surgery, TOF, Postoperative pulmonary complications

## Abstract

**Background:**

Postoperative delirium (POD) is a critical complication in patients accepting colon carcinoma surgery. Neostigmine, as a cholinesterase inhibitor, can enhance the transmission of cholinergic transmitters in synaptic space, and play an important role in maintaining the normal level of cognition, attention and consciousness. The objective of this study was to investigate the effect of neostigmine on POD and clinical prognosis.

**Methods:**

A randomized, double-blind controlled trial was implemented in Qingdao Municipal Hospital Affiliated to Qingdao University. A total of 454 patients aged 40 to 90 years old accepted colon carcinoma surgery were enrolled between June 7, 2020, and June 7, 2021, with final follow-up on December 8, 2021. Patients were randomly assigned to two groups: the neostigmine group (group N) and the placebo group (group P), the patients in group N were injected with 0.04 mg/kg neostigmine and 0.02 mg/kg atropine intravenously. The primary endpoint was the incidence of POD, researchers evaluated the occurrence of POD by the Confusion Assessment Method (CAM) twice daily (at 10 a.m. and 2 p.m.) during the first 7 postoperative days, POD severity was assessed by the Memorial Delirium Assessment Scale (MDAS). The secondary endpoints were the extubating time, postanesthesia care unit (PACU) time, the incidence of various postoperative complications, length of hospital stays, and 6 months postoperative mortality.

**Results:**

The incidence of POD was 20.20% (81/401), including 19.39% (38/196) in group N and 20.98% (43/205) in group P. There was no significant statistical significance in the incidence of POD between group N and group P (*P* > 0.05); Compared to group P, the extubating time and PACU time in group N were significantly reduced (*P* < 0.001), the incidence of postoperative pulmonary complications (POPCs) decreased significantly in group N (*P* < 0.05), while no significant differences were observed in postoperative hospital stay and mortality in 6 months between the two groups (*P* > 0.05).

**Conclusion:**

For patients accepted colon carcinoma surgery, neostigmine did not significantly reduce the incidence of POD, postoperative mortality and postoperative hospital stay, while it indeed reduced the extubating time, PACU time and the incidence of POPCs.

**Trial registration:**

The randomized, double-blind, controlled trial was registered retrospectively at www.chictr.org.cn on 07/06/2020 (ChiCTR2000033639).

## Background

Postoperative delirium (POD) is a common postoperative complication, which usually characterizes an acute decline in the patient’s cognitive state, attention and mental level, it is often starts in the recovery room and occurs up within one week after the surgery even later [1, 2]. POD is a syndrome with many influencing factors, anesthesia and surgical factors play a key role in its occurrence and development, On the other hand, the influence of patients’ demographic factors should not be underestimated. The incidence of POD varies widely in different situations, according to statistic, the occurrence of POD is between 4 and 65%, which is more common in elderly patients [3]. A systematic review and meta-analysis that analyzed 12 studies of POD after colorectal surgery found 15% elderly patients over 65 develop POD [4]. Though the precise etiology of POD is yet to be understood, the underlying biological bases is believed to be hypofunction of the cholinergic system within the central nervous system [5]. Cholinergic system is one of the most important regulatory neurotransmitter systems in the brain. Inhibition of acetylcholine M1 receptors in the postsynaptic membrane can lead to cognitive impairment such as hallucinations and confusion, meanwhile, acetylcholine can inhibit IL-6, IL-8 and TNF- α, it suggests that cholinergic system can protect brain tissue from inflammatory reaction [6]. Researches have shown that the levels of ACh in plasma and cerebrospinal fluid are low in patients with POD, this may be the result of neuroinflammatory response and synaptic damage [5, 7]. In addition, the use of a variety of anticholinergic drugs has been shown to increase the risk of delirium [8]. However, to date, there is still lack of evidence to prove that cholinergic system agonists, such as cholinesterase inhibitor, can reduce the incidence of POD.

Neostigmine is a cholinesterase inhibitor, which is used to treat myasthenia gravis and intestinal paralysis after abdominal surgery. In general anesthesia, Neostigmine is often used to antagonist the effects of nondepolarizing muscular relaxants. The positively charged nitrogen atom in neostigmine molecule can electrostatically combine with the negatively charged catalytic site of cholinesterase, and the carbamate group in the molecule is covalently combined with the enzymatic hydrolysis site of the enzyme, which is the carbamate of cholinesterase, so as to inhibit the activity of the enzyme. In addition, it can also directly excite the postsynaptic acetylcholine receptor or increase acetylcholine release by inhibiting potassium channel to promote muscle fiber contraction [9]. From this we can know that neostigmine can increase the level and duration of acetylcholine, so as to enhance the activity of cholinergic system in our brain.

Therefore, we intended to conduct a randomized, double-blind controlled trial to investigate the effect of neostigmine on POD and clinical prognosis in patients aged 40–90 years undergoing colon carcinoma surgery.

## Methods

### Participants

This study includes 445 qualified patients who were between 40 and 90 years of age and scheduled to have colon carcinoma surgery was performed under general anesthesia with endotracheal intubation combined with transversus abdominis plane (TAP) block, between June 7, 2020, and June 7, 2021, in Qingdao Municipal Hospital affiliated to Qingdao University. The inclusion criteria of this study contain (1) age 40–90 years; (2) Han Nationality Patients in north China; (3) American Society of Anesthesiologists (ASA) Grade I–III; (4) preoperative cognitive status was good with no language communication disorder; (5) educational level was enough to complete preoperative cognitive function test. The exclusion criteria contain (1) preoperative delirium, Parkinson’s disease, dementia caused by various reasons (including Parkinson’s disease related dementia, Alzheimer’s disease related dementia, Lewy body dementia), or major psychological dysfunction; (2) central nervous system infection, head trauma, stroke, epilepsy, multiple sclerosis and other major neurological diseases; (3) preoperative Mini-mental State Examination (MMSE) scores of 23 or less; (4) contraindications and allergic history of neostigmine, history of glaucoma or bromide allergy and genetic family history, liver and kidney dysfunctions; (5) taking sedatives, analgesics, or antidepressants; (6) unwillingness to comply with the protocol or procedures.

### Sample size calculation

In our study, we used PASS 11.0 (NCSS, LLC. Kaysville, Utah, USA) software to estimate the sample size required for the experiment, assuming a sensitivity of 0.9, a sensitivity tolerance of 0.05, a specificity tolerance of 0.05, α = 0.05, 1–β = 0.8, a bilateral, and a 20% dropout rate. Our preliminary experimental results showed that the incidence of postoperative delirium in patients who did not use neostigmine during surgery was 20.6%. In addition, we hypothesized that the use of neostigmine during surgery would reduce the incidence of POD by half. According to the sample size ratio of the experimental group and the control group is 1:1, the overall sample size was calculated as 454, each group contained 227 patients.

### Grouping and administration of neostigmine

The experimental designer first generated unrestricted random numbers without restriction (simple randomization) through the computerized system, sealed these random numbers in sequentially numbered envelopes, and sent them to the anesthesiologists of our research team by a research nurse the day before the surgery. At the same time, they were informed the group assignment, and upon consent of the selected patients, which were assigned to study groups based on random numbers. An allocated random number was used to perform block randomization in a 222:223 ratio. In Operation room, neostigmine (1 mg/2 ml, Batch Number: 2130504, Shanghai Xinyi Jinzhu Pharmaceutical Co., Ltd.), atropine or identical saline volume was administered to the patients according to the recovery of TOFR by the anesthesiologist. The dynamic changes of TOFR was monitored by TOF-GUARD INM type acceleration muscle relaxation tester (TOF-Watch SX, Organon, Ireland) immediately when the intravenous injection of muscle relaxant was discontinued. Monitor the contractile response of adductor pollicis muscle to judge the degree of muscle relaxation mainly depends on the stimulation of ulnar nerve through a transducer converter (TOF-Watch SX, Organon, Ireland). The parameters were set as TOF mode, current intensity 60 mA, with four series stimulations every 13 s. When TOF count ≥ 2 or the patients began to breathe autonomously, the patients in group N were injected with 0.04 mg/kg neostigmine intravenously and 0.02 mg/kg atropine, the patients in group P were intravenously injected with an identical saline volume.

### Anesthesia and surgery

Before the surgery, all patients strictly observed at least 6-h fasting and 2-h water fasting without any preoperative medication. None of the enrolled patients received any sedative or analgesic treatment prior to induction of anesthesia. Once the patient entered the operation room, after the third-party verification, the anesthesiologist used a Drager monitor (model: Primus, Qingdao unity medical co.) to collect Vital parameters immediately, such as oxygen saturation (SpO2), invasive radial arterial pressure, non-invasive arterial blood pressure (NABP), heart rate (HR), electrocardiogram (ECG), and end-tidal carbon dioxide partial pressure (PETCO2), body temperature. Moreover, a disposable bispectral index (BIS) sensor was applied to the patient’s forehead after the skin was wiped with an alcohol swab, which was connected with BIS monitor (the Germany, Philips, M1034A Co.) to monitor the depth of anesthesia of patient. Provide 100% oxygen to the mask before intubation.

We chose 0.2 mg/kg etomidate, 0.5 μg/kg sufentanil, and 0.2 mg/kg cisatracurium besylate were used for rapid intravenous induction. Endotracheal intubate was performed using a visual laryngoscope. 7.5# endotracheal tube was selected for male patients and 7.0# endotracheal tube for female patients. After intubation, the parameters of the anesthesia machine were set as tidal volume 8–10 ml/kg, respiratory rate 8–12 time/min, airway pressure < 30 mmHg, and partial pressure of carbon dioxide during expiration between 35 and 45 mmHg. Radial artery and internal jugular vein catheterization were performed under ultrasound guidance to monitor invasive arterial pressure and central venous pressure (CVP). All patients underwent TAP block (40 ml, 0.375% ropivacaine) to reduce postoperative pain.

Anesthesia was kept with propofol (6–8 mg/kg/h), remifentanil (0.1–0.3 μg/kg/min), sevoflurane (1–2%), and cisatracurium besylate (0.1–0.2 mg/kg/h). Maintain the fluctuation range of the patient’s average arterial pressure (MAP) and heart rate within 20% of the basic value, otherwise vasoactive drugs shall be used according to the actual situation. When the MAP was below 20% of the baseline value, 6 mg/time ephedrine was administrated, whereas when it was above 20% of the baseline value, 5–10 mg urapidil was administered. When the heart rate was above 90 beats/minute, 1 mg/kg esmolol was administered, whereas when it was below 50 beats/minute, 0.3 mg atropine was administered. Closely observe the changes of blood pressure and heart rate after treatment, and take further treatment if necessary. Glucocorticoid drugs, dexmedetomidine nonsteroidal analgesics, and midazolam were avoided during surgery. The intraoperative TOF count was maintained at 0. BIS value was maintained between 40 and 60. The axillary temperature was maintained between 36.0 °C and 37.4 °C. The intraoperative intravenous solute was set at 6–8 ml/kg/h.

Sevoflurane inhalation was terminated about 20 min before the end of surgery, and the intravenous injection of cisatracurium besylate, propofol, remifentanil was discontinued at the beginning of skin closure. The neostigmine and atropine were intravenously injected based on the TOF count or the condition of spontaneous breathing in group N. The patients in group P were intravenously injected with an identical saline volume. Ensure that all patients were extubated with a TOF ratio > 0.9 and transferred to PACU 10 min after extubation, an analgesic pump was connected for patient-controlled intravenous analgesia at the same time (PCIA) (8 to 12 mg butorphanol, 5 mg tropisetron were added into 100 ml saline, infusion dose: 2 ml/hour, demand dose: 0.5 ml/time, lock time: 15 min). The vital signs of the patients were closely observed in the PACU. When the BIS value exceeded 90, and the Steward resuscitation score was ≥ 6 points, the patients were sent to the ward.

### Study outcomes and other observation indexes

The primary endpoint of this study was the incidence of POD on 1–7 days (or before discharge). The occurence of POD was evaluated by the Confusion Assessment Scale (CAM) [10], which using the standards formulated by the diagnostic and Statistical Manual of mental diseases (Fourth Edition) (DSM-IV) of the American Psychiatric Association at 10 a.m. and 2 p.m. twice a day by an anesthesiologist post-operatively. The diagnosis of POD included the following four clinical criteria: (1) acute onset and fluctuation process; (2) inattention; (3) disorganized thinking; and (4) change of consciousness level. POD can be diagnosed if it meets the standards (1), (2), and (3) or (4) at the same time. As for POD severity, which was assessed immediately through the Memorial Delirium Assessment Scale (MDAS) followed CAM evaluation by the same visitor [11].

The secondary endpoints were the extubating time (time period from skin closure to tracheal tube extraction), PACU time, the incidence of various postoperative complications, such as postoperative pulmonary complications (POPCs, including pneumonia, atelectasis respiratory failure, and pulmonary embolism), abnormal muscle weakness, postoperative nausea and vomiting (PONV), gastrointestinal complications (abdominal tenderness, distention, and ileus), wound bleeding and infection, cardiac complications (arrhythmia, angina, myocardial infarction, and cardiac arrest) and cerebrovascular complications (cerebral hemorrhage and cerebral infarction), length of hospital stays, and 6 months postoperative mortality.

As for other observation indexes, the baseline data of patients were collected first, among them a neurologist used MMSE scale (full score 30 points, score > 23 points to be included; score ≤ 23 points to be excluded) [12] and Pittsburgh sleep quality index (PSQI) scale (The total score range is 0–21, the lower the score, the better the sleep quality) [13] to evaluate the cognitive function and sleep quality of the patients. The rest include clinical features of the patients during perioperative period, such as intraoperative mean arterial blood pressure, mean heart rate, mean blood oxygen saturation, and mean temperature, infusion volume, bleeding volume, urine volume, operation time, and anesthesia time.

### Blinding

The surgeons who participating in the operation, patients, and family members were blinded to group allocation. Bedside anesthesiologists involved in the operation were the only members of the research team aware of group allocation and were responsible for managing drug injection as per trial protocol. The preoperative evaluation was conducted by a neurologist, and the postoperative evaluation was conducted by an anesthesiologist, neither of the two researchers is participant in the patient’s intraoperative management, and do not consult the patient’s medical records and relevant examination results. Physicians performing preoperative or postoperative interview and assessments, statistics personnel, were blinded to group allocation, as were all members of postoperative treatment and nursing. All evaluators received a two-week unified training before the start of the study.

### Statistical analysis

The data analysis in this study adopted SPSS statistical software, version 21.0 (SPSS, Inc., Chicago, IL, USA), and GraphPad Prism software, version 6.01 (GraphPad Software, Inc., La Jolla, CA, USA). The Shapiro–Wilk test was used to assess the normality of continuous data. Meeting in line with the normal distribution of measurement data, were presented as mean ± standard deviation (‾x ± s), and independent sample t-test was used to compare between the groups. The measurement data of skew distribution are expressed by median (interquartile range) [M(Q)], and rank-sum test was used to compare between the groups. Count data between the two groups were compared with chi-square test. *P* < 0.05 was statistically significant.

## Results

### Participants’ demographic characteristics

**Fig. 1:**
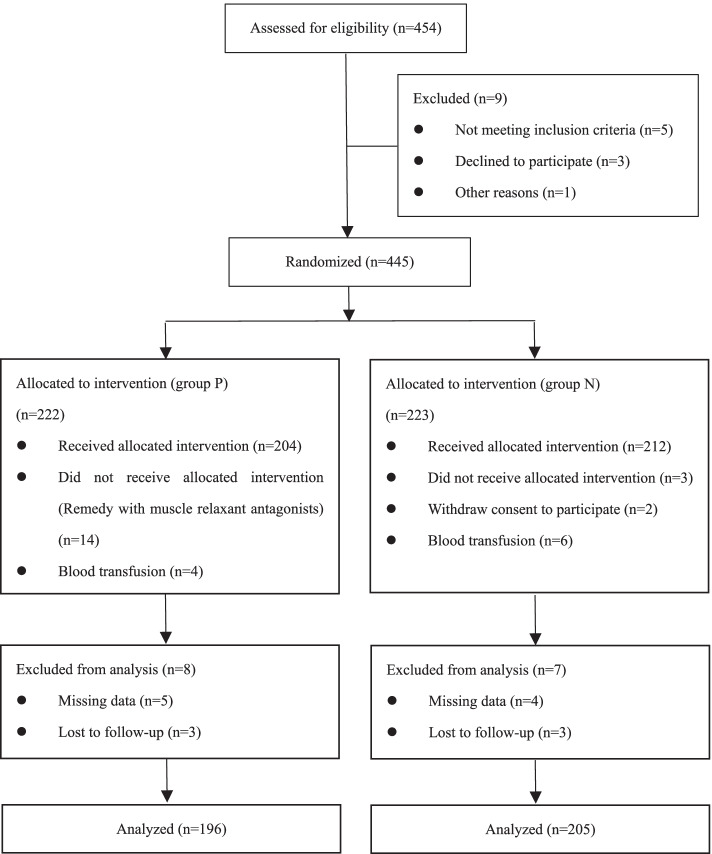
Flow chart of the trial

The present study enrolled 454 participants, including 3 people refused to participate. 222 and 223 participants were randomly divided into two groups: the neostigmine group (group N), the placebo group (group P). 53 participants were excluded. The criteria are shown in Fig. 1. Finally, 196 participants in group N and 205 participants in group P were included in the analysis. All patients underwent colon carcinoma surgery was performed under general anesthesia with endotracheal intubation combined with transversus abdominis plane (TAP) block.

### Basic characteristics of the patients among two groups

No significant differences in age, sex, height, body weight, ASA grade, underlying diseases such as hypertension, diabetes, coronary heart disease, drinking history, smoking history, preoperative Hb, preoperative albumin, years of education, preoperative MMSE score and preoperative PSQI score were observed among two groups in Table 1 (*P* > 0.05).

**Table 1:** Basic characteristics of patients among two groups

	Group N (*n* = 196)	Group P (*n* = 205)	*P*-value
Age (year, $\overline x$ ± s)	64.19 ± 10.51	63.34 ± 10.38	0.414
Sex, n (%)			0.834
Male, n (%)	106(54.08%)	113(55.12%)	
Female, n (%)	90(45.92%)	92(44.88%)	
Height (cm, $\overline x$ ± s)	165.47 ± 7.81	165.78 ± 7.54	0.695
Body weight (kg, $\overline x$ ± s)	66.95 ± 11.67	68.13 ± 11.43	0.309
ASA grade, n (%)			0.604
I, n (%) II, n (%)	11(5.61%) 156(79.59%)	11(5.37%) 156(76.10%)	
III, n (%)	29(14.80%)	38(18.54%)	
Hypertension n, (%)	90(45.92%)	93(45.37%)	0.912
Diabetes n, (%)	53(27.04%)	52(25.37%)	0.703
Coronary heart disease n, (%)	50(25.51%)	43(20.98%)	0.282
Drinking history n, (%)	90(45.92%)	94(45.85%)	0.990
Smoking history n, (%)	72(36.73%)	81(39.51%)	0.567
Preoperative Hb (g/L, x ± s)	127.81 ± 20.72	127.29 ± 20.98	0.802
Preoperative albumin (g/L, $\overline x$ ± s)	37.88 ± 4.04	37.84 ± 4.17	0.915
Years of education n, (%)			0.143
0 n, (%)	10(5.10%)	15(7.32%)	
1 to 6 n, (%)	52(26.53%)	50(24.39%)	
7 to 9 n, (%)	73(37.24%)	84(40.98%)	
10 to 12 n, (%)	56(28.57%)	43(20.98%)	
> 12 n, (%)	5(2.55%)	13(6.34%)	
Preoperative MMSE score (point, $\overline x$ ± s)	26.66 ± 1.34	26.72 ± 1.42	0.644
Preoperative PSQI score (point, $\overline x$ ± s)	7.43 ± 3.56	7.44 ± 3.58	0.966

Clinical characteristics of the patients among two groups during perioperative period.

**Fig. 2:**
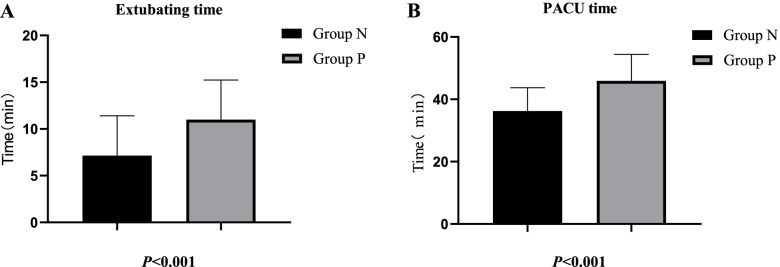
**A** Comparison of extubating time of patients in the two groups included. **B** Comparison of PACU time of patients in the two groups included

As shown in Table 2, intraoperative mean arterial blood pressure, mean heart rate, mean blood oxygen saturation, and mean temperature, infusion volume, bleeding volume, urine volume, operation time, anesthesia time and TOF ratio before extubation of the patients among two groups remained similar (*P* > 0.05). Nevertheless, we can find that the extubating time and PACU time in group N was significantly reduced (*P* < 0.001) compared to group P in Fig. 2.

**Table 2:** Clinical characteristics of patients among two groups during perioperative period

	Group N (*n* = 196)	Group P (*n* = 205)	*P*-value
Intraoperative mean arterial blood pressure (mmHg, $\overline x$ ± s)	86.34 ± 9.01	87.51 ± 8.95	0.192
Intraoperative mean heart rate (time/min, $\overline x$ ± s)	65.06 ± 6.45	65.00 ± 5.99	0.934
Intraoperative mean blood oxygen saturation (%, $\overline x$ ± s)	99.79 ± 0.68	99.80 ± 0.61	0.828
Intraoperative mean temperature (℃, $\overline x$ ± s)	36.28 ± 0.39	36.30 ± 0.38	0.477
Infusion volume (ml, $\overline x$ ± s)	1789.03 ± 756.32	1760.37 ± 811.86	0.417
Bleeding volume (ml, $\overline x$ ± s)	103.70 ± 97.37	100.24 ± 92.18	0.715
Urine volume (ml, $\overline x$ ± s)	421.33 ± 345.81	390.24 ± 312.41	0.207
Operation time (min, $\overline x$ ± s)	184.97 ± 82.01	187.34 ± 91.67	0.786
Anesthesia time (min, $\overline x$ ± s)	238.95 ± 93.70	240.76 ± 106.10	0.857
TOF ratio > 0.9 before extubation n, (%)	196(100%)	205(100%)	1.000

### Postoperative characteristics of patients among two groups

As listed in Table 3, there was no significant difference in the incidence and duration of POD, postoperative MDAS score and postoperative 24 h NRS score among two groups (*P* > 0.05). Compared to group P, the incidence of pulmonary complications decreased significantly in the neostigmine group (*P* = 0.034), while no obvious differences were observed in terms of the incidence of abnormal muscle weakness, PONV, gastrointestinal complications, wound bleeding and infection, cardiac complications and cerebrovascular complications, postoperative hospital stay and mortality in 6 months between the group N and group P (*P* > 0.05).

**Table 3:** Postoperative characteristics of patients among two groups

	Group N (*n* = 196)	Group P (*n* = 205)	*P*-value
POD, n (%)	38(19.39%)	43(20.98%)	0.692
POD duration n, (%)			0.928
Non-POD n, (%)	158(80.61%)	162(79.02%)	
0 to 6 h n, (%)	22(11.22%)	26(12.68%)	
6 to 12 h n, (%)	9(4.59%)	11(5.37%)	
> 12 h n, (%)	7(3.57%)	6(2.93%)	
Postoperative MDAS score (point, $\overline x$ ± s)	8.17 ± 3.44	8.36 ± 3.37	0.571
Postoperative 24 h NRS score (point, $\overline x$ ± s)	1.98 ± 1.29	2.06 ± 1.22	0.505
Abnormal muscle weakness, n (%)	0(0%)	0(0%)	
PONV, n (%)	24(12.24%)	22(10.73%)	0.635
Gastrointestinal complications, n (%)	35(17.86%)	48(23.41%)	0.170
Wound bleeding and infection, n (%)	13(6.63%)	17(8.29%)	0.528
POPCs, n (%)	42(21.43%)	63(30.73%)	0.034*
Cardiac complications, n (%)	3(1.53%)	2(0.98%)	0.679
Cerebrovascular complications, n (%)	1(0.51%)	2(0.98%)	1.000
Postoperative hospital stay (day, $\overline x$ ± s)	12.44 ± 2.78	12.54 ± 2.50	0.726
Mortality in 6 months, n (%)	3(1.53%)	4(1.96%)	1.000

*Abbreviation*: *PONV* Postoperative nausea and vomiting, *POPCs* Postoperative pulmonary complications

^*^P < 0.05 in comparison with group P

## Discussion

In this randomized, double-blind controlled trial, we assessed the effect of neostigmine on POD in patients undergoing colon carcinoma surgery by CAM and MDAS, collected clinical characteristics during perioperative period and postoperative characteristics of patients among two groups to evaluate clinical prognosis of patients. Ultimately, the total incidence of POD among the two groups was 20.20% (81/401), the incidence of POD in group N was 19.39% (38/196), and the incidence of POD in group P was 20.98% (43/205), it shows that neostigmine has no specific effect on the occurrence of POD. The secondary outcomes were similar between the two groups, except that the incidence of POPCs in group N was relatively low, the extubation time and PACU time in group N were significantly shorter than those in group P.

The central cholinergic system may be the essential neurotransmitter system regulating memory, attention, and learning [14]. Current evidence shows that cholinergic system damage is one of the important reasons for the occurrence and development of neurodegenerative diseases, and the most distinctive feature is amyloid β peptide abnormal deposition and apoptosis of cholinergic neurons in forebrain [15, 16]. The severe loss of pre basal cholinergic neurons and the decrease of choline acetyltransferase level weaken the control of hippocampus and neocortex, damage the activation mechanism of cerebral cortex, resulting in the progressive decline of cognitive condition and disturbance of behavior [15, 17]. At present, it has been confirmed that the cholinesterase inhibitor such as rivastigmine can be used to improve the cognitive function of patients with neurodegenerative diseases by enhancing cholinergic neurotransmission in the brain [18]. Meanwhile, the cholinergic system plays an important role in the anti-inflammatory pathway [19], rat experiments showed that neostigmine could reduce IL-1β in cortex and hippocampus, reduce the gene expression of proinflammatory cytokines and the activity of acetylcholinesterase, which may reduce and delay the proinflammatory response and neurodegeneration of cerebral cortex and hippocampus after surgery [20]. On the other hand, studies have shown that cholinesterase, as one of the components of senile plaques, stimulate the assembly of amyloid fibers and combine with them to format highly toxic Aβ–AChE complexes which have a neurotoxic effect higher than that of both alone [21]. Therefore, substances that inhibit cholinesterase may have potential of neuroprotective agents. Neostigmine, as the most widely used cholinesterase inhibitor in general anesthesia, can often antagonize the residual muscle relaxation after surgery, which has naturally become the most appropriate intervention factor for our research. Recently, a randomized controlled trial of 120 patients undergoing the radical section of gastrointestinal tumors showed that the incidence of early postoperative cognitive decline in elderly patients was significantly reduced when quantitative neostigmine was routinely used after surgery, but may not be closely related to the changes of peripheral inflammatory factors. Unexpectedly, in our study, we did not find that neostigmine could reduce the incidence of POD.

At present, several reviews considered that the existing evidence is not enough to recommend cholinesterase inhibitors for the prevention or treatment of delirium in the elderly [22–24]. Batistaki et al. found that neostigmine / atropine to reverse the residual muscle relaxation caused by rocuronium did not affect the incidence of POCD after elective surgery and general anesthesia [25], which is similar with the conclusion of our study. On the other hand, neostigmine is more used to antagonize the residual muscle relaxation after general anesthesia, however, the antagonistic effect of neostigmine on neuromuscular block can only be carried out in the presence of evidence of autonomous recovery of muscle strength, researches confirmed that early administration of neostigmine cannot shorten the overall recovery time and is not beneficial to clinic [26]. Clinical experiments have shown that with the gradual reduction of degree of neuromuscular block, the dose and the mean reversal time of neostigmine are also reduced [26]. On the contrary, when neuromuscular function is fully restored, the use of anticholinesterase drugs may lead to abnormal muscle weakness [27]. Therefore, it is very important to choose an appropriate timing of administration, we choose TOF count ≥ 2 or the patients began to breathe autonomously as the time of neostigmine administration in our study. In addition, neostigmine is decomposed mostly by acetylcholinesterase at the neuromuscular junction and kidney, and the time of neostigmine reaching the peak effect is 7–11 min, the elimination half live is 77 min. From this, it shows that neostigmine is mainly used after operation, and the action time is short. Therefore, neostigmine may not eliminate the damage of anesthesia and surgical factors to the central nervous system, and reverse the existing trend of the decline of cholinergic system function in such a short time. Moreover, agonists of central nAChRs and mAChRs may improve the performance of cognitive, attention and consciousness levels, while antagonists will damage the corresponding functions [28]. Although the effect pattern of anticholinesterase drugs on cholinergic receptors is still unclear, studies have shown that pyridostigmine activates M1 and M3 receptor, but physostigmine activates only the M1 receptor and neostigmine activates only M3 [29]. This may also be one of the reasons for no obvious effect of neostigmine in improving postoperative cognitive status.

Neostigmine can increase the content of acetylcholine in synaptic space, activate nAChRs, promote neuromuscular excitation transmission and reverse muscle relaxation. At the same time, controlled by autonomic postganglionic fibers, mAChRs is activated at the same time, which may cause a series of adverse reactions. Anticholinergic drugs such as atropine can relieve the obvious vagal effect produced by cholinesterase inhibitors, prevent the occurrence of bradyarrhythmia, directly dilate bronchus and reduce the risk of bronchospasm. In addition, atropine can also penetrate through the blood–brain barrier and affect the central nervous system to reduce the occurrence of PONV, hence the patients in group N received atropine routinely after injection of neostigmine. In our study, the incidence of abnormal muscle weakness, PONV in group N did not increase significantly, and compared with group P, the extubating and PACU time was reduced without prolonging postoperative hospital stay, which is similar to the results of previous studies [30].

Residual neuromuscular block after general anesthesia has been associated with airway obstruction, pneumonia, atelectasis, and respiratory failure, particularly in older patients, and will inevitably increase length of stay and cost of patients [31–33]. In a large retrospective study conducted in the Netherlands, researchers found that patients who used a non-depolarizing neuromuscular blocking drug (NDNMBD) during surgery had a higher risk of POPCs, and patients who used NDNMBD but did not use reversal agents were 2.3 times more likely to develop POPCs than patients who received neostigmine [33]. Residual neuromuscular block after neostigmine reversal has been still seen as the gold standard after general anesthesia at present. In our study, group P did not use reversal agents, all patients were extubated with a TOF ratio > 0.9 and monitored in PACU strictly to avoid the occurrence of related adverse events. Nevertheless, our results still show that postoperative intravenous neostigmine did significantly reduce the incidence of POPCs in patients after colon carcinoma surgery. Nevertheless, evidence had emerged showing that neostigmine can produce nerve block effect in individuals whose neuromuscular function has been completely restored [27]. For instance, when the neuromuscular function is normal, intravenous neostigmine will reduce the expandable volume of the upper respiratory tract, damage the function of genioglossus muscle and diaphragm, and increase the risk of postoperative adverse respiratory events [34]. We therefore conclude that proper monitor of neuromuscular blockade and the judicious use of muscle relaxant antagonist are important components in the care of postoperative patients and preventing POPCs. As for strategies, such as neuromuscular monitor was routinely used to judge the recovery of block, would likely be best prevention in current clinical practice.

This study has several limitations. First, obviously, it is a single-center study, only patients with colon carcinoma surgery were included, more types of surgeries and multi-center studies are needed to confirm the results of this study. Second, the patients in the experimental group were injected with 0.04 mg/kg neostigmine intravenously, we did not assess the effects of different injection concentrations of neostigmine on postoperative cognition and body recovery. Third, we did not assess the neurocognitive status of patients after discharge, six months or even one year, during this period, patients may also have corresponding cognitive level fluctuations, which will affect our evaluation results.

In conclusion, although there was no significant effect of neostigmine on POD in patients aged 40–90 years undergoing colon carcinoma surgery, the timely use of 0.04 mg/kg neostigmine after surgery can obviously accelerate patients’ recovery and decrease the incidence of POPCs.

## Data Availability

The raw data supporting the conclusions of this article from the study will be made available by the corresponding authors, without undue reservation. All the raw data included basic characteristics, clinical characteristics and postoperative characteristics of patients, are attached in supplementary file "standard data" and have been submitted with the article.

## Abbreviations

POD: Postoperative delirium
CAM: Confusion Assessment Method
MDAS: Memorial Delirium Assessment Scale
MMSE: Mini-mental State Examination
PSQI: Pittsburgh sleep quality index
POPCs: Postoperative pulmonary complications
PONV: Postoperative nausea and vomiting
PACU: Postanesthesia care unit
TAP: Transversus abdominis plane
ASA: American Society of Anesthesiologists
SpO2: Oxygen saturation
NABP: Non-invasive arterial blood pressure
HR: Heart rate
ECG: Electrocardiogram
PETCO2: End-tidal carbon dioxide partial pressure
BIS: Bispectral index
CVP: Central venous pressure
MAP: Average arterial pressure
